# Developing Hypothetical Inhibition Mechanism of Novel Urea Transporter B Inhibitor

**DOI:** 10.1038/srep05775

**Published:** 2014-07-22

**Authors:** Min Li, Weng Ieong Tou, Hong Zhou, Fei Li, Huiwen Ren, Calvin Yu-Chian Chen, Baoxue Yang

**Affiliations:** 1 The State Key Laboratory of Natural and Biomimetic Drugs, Department of Pharmacology, School of Basic Medical Sciences, Peking University, Beijing, 100191, China; 2 School of Medicine, College of Medicine, China Medical University, Taichung, 40402, Taiwan; 3 School of Pharmaceutical Sciences, Hubei University of Medicine, Shiyan, 442000, China; 4 Human Genetic Center, Department of Medical Research, China Medical University Hospital, Taichung, Taiwan; 5 Department of Biomedical Informatics, Asia University, Taichung, 41354, Taiwan; 6 Research Center for Chinese Medicine & Acupuncture, China Medical University, Taichung 40402, Taiwan; 8 These authors contributed equally to this work.

## Abstract

Urea transporter B (UT-B) is a membrane channel protein that specifically transports urea. UT-B null mouse exhibited urea selective urine concentrating ability deficiency, which suggests the potential clinical applications of the UT-B inhibitors as novel diuretics. Primary high-throughput virtual screening (HTVS) of 50000 small-molecular drug-like compounds identified 2319 hit compounds. These 2319 compounds were screened by high-throughput screening using an erythrocyte osmotic lysis assay. Based on the pharmacological data, putative UT-B binding sites were identified by structure-based drug design and validated by ligand-based and QSAR model. Additionally, UT-B structural and functional characteristics under inhibitors treated and untreated conditions were simulated by molecular dynamics (MD). As the result, we identified four classes of compounds with UT-B inhibitory activity and predicted a human UT-B model, based on which computative binding sites were identified and validated. A novel potential mechanism of UT-B inhibitory activity was discovered by comparing UT-B from different species. Results suggest residue PHE198 in rat and mouse UT-B might block the inhibitor migration pathway. Inhibitory mechanisms of UT-B inhibitors and the functions of key residues in UT-B were proposed. The binding site analysis provides a structural basis for lead identification and optimization of UT-B inhibitors.

Urea transporter B (UT-B) is a membrane protein extensively expressed in various tissues, such as kidney, testis, brain, bone marrow, spleen and erythrocyte^1^,^2^,^3^. Its physiological function has been well studied in kidney^4^,^5^,^6^.UT-B is expressed in endothelia of kidney descending *vasa recta* (DVR) and mediates the passive transport of urea down its concentration gradient, indispensably in renal urea recycling and urine concentration^7^,^8^. UT-B null mice exhibited urine output approximately 50% higher, and urine osmolality approximately 1/3 lower than in wild-type mice^9^,^10^, which implies that UT-B plays an important role in urinary concentrating ability and suggests the clinical applications of UT-B inhibitors as potential novel diuretics^11^,^12^,^13^,^14^,^15^,^16^,^17^,^18^. Recently, determination of the *Bos taurus* (Bovine) UT-B X-ray crystal structure provided a foundation for UT-B binding site identification and inhibitor discovery^19^,^20^.

To exploit novel compounds with UT-B inhibitory activity and to obtain promising lead compounds, we integrated cell based high throughput screening and *in silico* methods to identify a new potential UT-B inhibitor binding site and proposed the mechanism of UT-B inhibitor in different species. A small-molecule drug-like compound library of 50000 compounds was screened by high-throughput virtual screening (HTVS), which produced 2319 primary hit compounds for UT-B inhibitor. Then we employed a medium-throughput screening using an erythrocyte osmotic lysis assay and identified 4 compounds, PU_21_, PU_168_, PU_468_ and PU_474_, with UT-B inhibitory activity *in vitro* from the 2319 hits. 16 compounds with UT-B inhibitory activity were screened by erythrocyte osmotic lysis assay from 60 analogues of PU_21_ [REN et al., under review]^21^. PU_14_, one of the 16 compounds, exhibited potential inhibition activity in human, rabbit, rat, mouse *in vitro* and pharmacological diuresis activity *in vivo*^21^. Based on the physiological data, we built a computational mode of human UT-B by homology modeling. The putative UT-B binding sites were identified by structure-based drug design and validated by ligand-based and QSAR model. Additionally, UT-B structural and functional characteristics under inhibitors treated and untreated conditions were simulated by molecular dynamics (MD) simulation. The UT-B inhibitor binding site analysis and validation provide a structural basis for lead identification and optimization.

## Results

### UT-B inhibitors identified by HTVS

**Figure 1:**
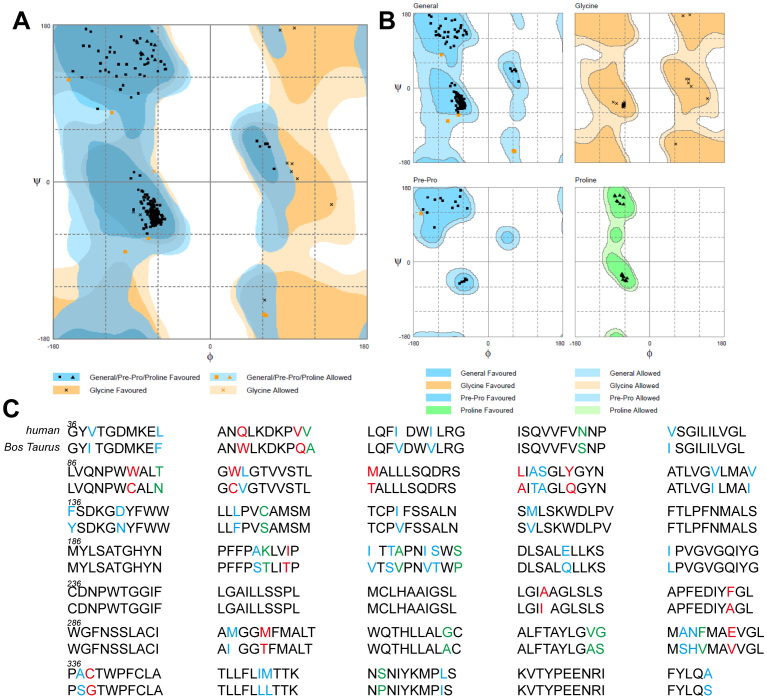
(a, b) Ramachandran plot assessment validation of predicted human UT-B structure. (c) Sequence alignment results: Human sequence compared with *Bos Taurus*.

**Figure 2:**
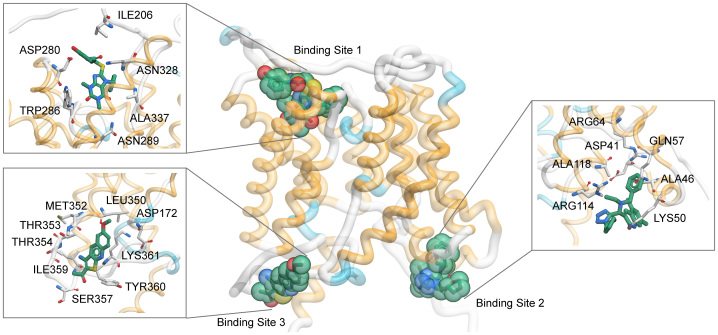
One extracellular (binding site 1) and two intracellular (binding site 2 and 3) hypothetic binding sites are predicted. Key residues that are predicted to interact with UT-B inhibitors are shown. Pocket residues are shown in white while the distance between residue and ligand was set to 2.5Å. Cross-species and mutation studies suggest binding site 1 might be a possible pocket for UT-B inhibitor binding (see below).

Sequence alignment was used to assess the suitability for homology modeling. The results of multiple sequence alignment show an 83.8% sequence identity and 92.8% sequence similarity between bovine and human UT-B. The Ramachandran plot shows that seven residues were distributed in the allowed region, including ASN73, ASN89, PHE176, THR191, GLY298, CYS338 and THR368 (Figure 1). A total of 336 residues were distributed within the region. Residues V206I, H328N, and S337A were involved in the binding site of the UT-B ligand, which suggests that species-specific differences of the UT-B binding site may influence the ligand binding affinity. One observable extracellular binding site (binding site 1) and two intracellular binding sites (binding sites 2 and 3) were predicted for HTVS (Figure 2). One cross-helix loop was located upside binding site 1 and urea binding site. Binding site 2 was bound in α-helix, β-turn and loop whereas binding site 3 was located between three α-helices. Based on the three binding sites, 2,319 compounds were identified by HTVS from 50,000 small-molecular drug-like compounds in Asinex database.

### Identification of small-molecular UT-B inhibitors by screening

**Figure 3:**
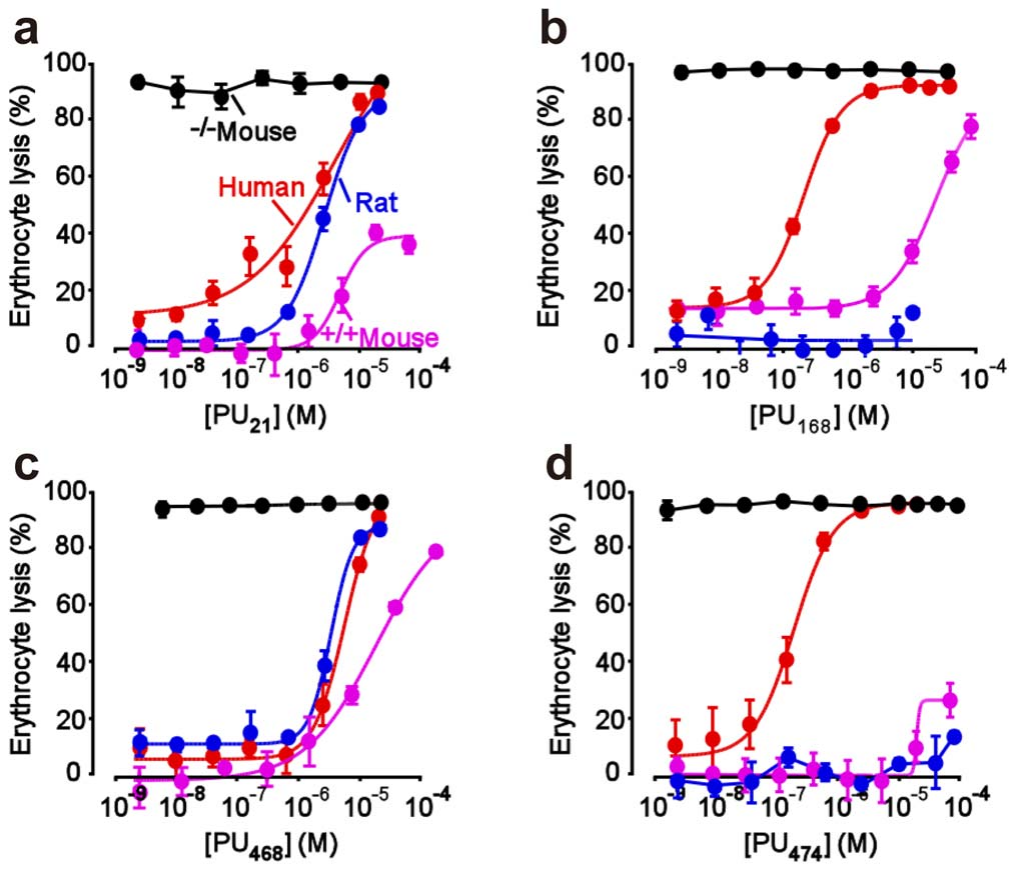
Activity of inhibitors against UT-B in human, rat and mouse. (a) Dose-dependent inhibition activity for PU_21_, determined by the osmotic lysis assay in human,rat and mouse erythrocytes. (b) Dose-dependent inhibition activity for PU_168_. (c) Dose-dependent inhibition activity for PU_468_. (d) Dose-dependent inhibition activity for PU_474_. Mean ± s.e.m., n = 3.

Four compounds, [1-(3-amino-6-methoxythieno[2,3-b]quinolin-2-yl)ethanone], [3-((R)-(benzyl(ethyl)amino)(1-(((S)-tetrahydrofuran-2-yl)methyl)-1H-tetrazol-5-yl) methyl)-5,7-dimethylquinolin-2(1H)-one], [N-(3-(4-chlorobenzyl)-2-methyl-4- oxo-3,4-dihydroquinazolin-6-yl)furan-2-carboxamide], and [2-((7-benzyl-1,3-dimethyl- 2,6-dioxo-2,3,6,7-tetrahydro-1H-purin-8-yl)thio)-N-(3-hydroxyphenyl)acetamide] (PU_21_, PU_168_, PU_468_, PU_474_) exhibiting UT-B inhibition activity were identified from 2319 candidate compounds by an erythrocyte osmotic lysis assay. As the results show, human UT-B is more sensitive to active compounds than UT-B from two other species. Inhibition activity of each compound on human, rat, and mouse are shown in Figure 3. The IC_50_s for each compound are summarized in Table 1.

### Structure-based drug design

**Figure 4:**
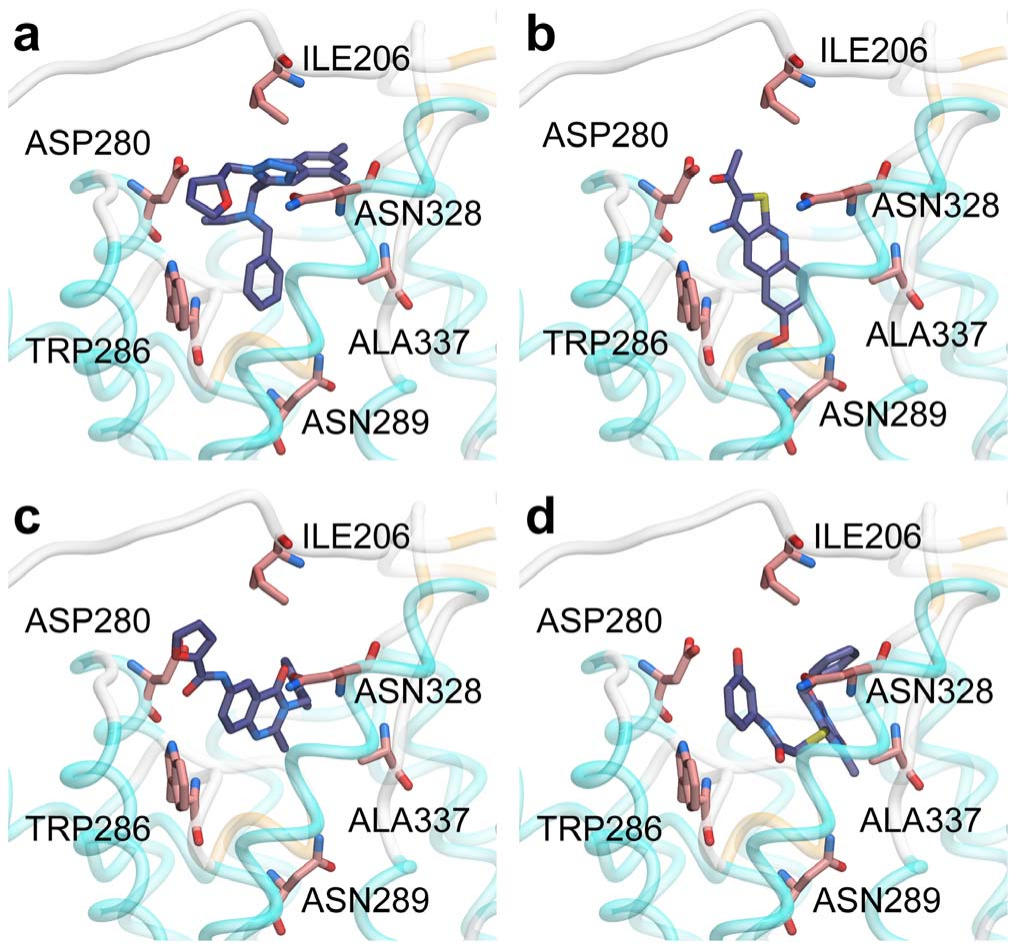
Binding pose of (a) PU_168_, (b) PU_21_, (c) PU_468_, (d) PU_474_ in the UT-B predicted binding site. Rigid docking pose provides details of the functional group-residue interactions and insights into residue mutation. The hydroxyl group of PU_21_ can interact with ASN289 inside the binding pocket whereas PU_168_, PU_468_ and PU_474_ cannot.

Based on the high sequence identity and similarity, we proceeded to model human UT-B structure using bovine UT-B structure. Candidate selection and ligand affinity was primarily based on Dock score, which combines the protein-ligand energy and ligand internal energy. The docking pose and Dock score of compounds PU_21_, PU_168_, PU_468_ and PU_474_ are shown in Table 2 and Figure 4. Type and occurrence of ligand-residual interaction is defined as important criterion for evaluating the function of residues. PU_168_ forms π–π interactions with TRP286 and hydrogen bonds with ALA337 which are anchored in binding site (Figure 4a). PU_21_ anchors in the binding site through generating hydrogen bonds with ASN289 (Figure 4b) whereas PU_468_ failed to generate hydrogen bonds and π–π interactions with any residue, but generated Van der Waals forces and hydrophobic interactions with LEU285 and ALA327 (Figure 4c). PU_474_ forms more hydrogen bonds than the other compounds with ILE206, ASP280, ASN289 and ASN328. PU_474_ also generates π–π interactions with TRP286 (Figure 4d). On the other hand, residues LEU285 and ALA327 generate strong Van der Waals forces and hydrophobic interactions with PU_21_, PU_168_, PU_468_ and PU_474_.

**Figure 5:**
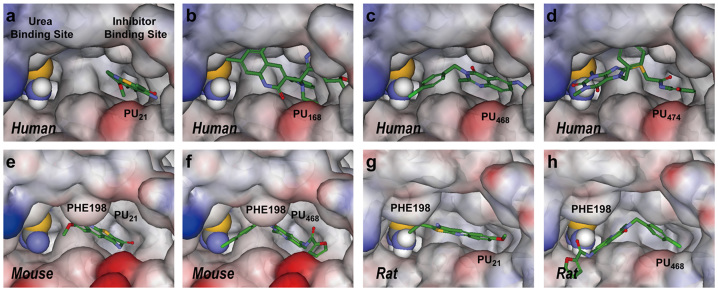
Figure illustrates the structural differences in inhibitor binding site in UT-B between human, mouse and rat. Re-docking poses of PU_21_, PU_168_, PU_468_ and PU_474_ in human are shown at (a–d), respectively. PU_21_ and PU_468_ re-docking poses in mouse and rat UT-B are shown at (e–f) and (g–h), respectively. The tertiary structure of the extracellular binding site demonstrates the steric hindrance difference, which is caused by PHE198 in mouse or rat UT-B. Only PU_21_ and PU_468_ can anchor in human, mouse and rat UT-B.

In a mutation study, PU_168_ docking with a W286G model showed a significant increase in binding affinity, from 19.037 to 55.154. The binding affinity of PU_474_ is also enhanced in the W286G model, with an increase to 5.778, suggesting TRP286 might have a weak binding affinity function. Residues of inhibitor binding site include ILE206, VAL324, ASN328 and ALA337 in human, which are different from other species. However, the binding affinity residuals of PU_21_, PU_168_, PU_474_ in rat and mouse UT-B show a significant increase compared with UT-B in human (Table 3). Homology models superimposed by multiple sequence alignments show that the extracellular loop of mouse and rat UT-B folded to form a different structure compared with human UT-B, with a RMSD of 0.1411 Å and 0.1114 Å, respectively (Table 4). In addition, there are high folding distances between human: rat and human: mouse of 15.8 Å and 18.1 Å, respectively. Insight from fined-grained view, residue PHE198 of rat and mouse UT-B, located in the extracellular flexible loop, generate a steric bulk that evolves into the formation of an inhibitor binding site (Figure 5). The results of the binding affinity calculation and the formation of PHE198 suggest that PHE198 might block the inhibitor migration pathway.

### Ligand-based drug design

The genetic function approximation (GFA) that generated a model with a coefficient of determination (R^2^) of 0.8188 was used to determine the most representative descriptors: ES_Sum_ssCH2, ES_Sum_aaCH, ES_Sum_dssC, ES_Sum_aasC, ES_Count_dssC, Molecular_Solubility, Num_H_Acceptors, Molecular_PolarSurfaceArea, Molecular_PolarSASA, Energy (Table S1). 
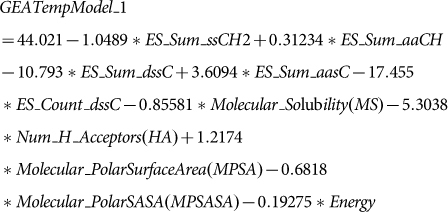


**Figure 6:**
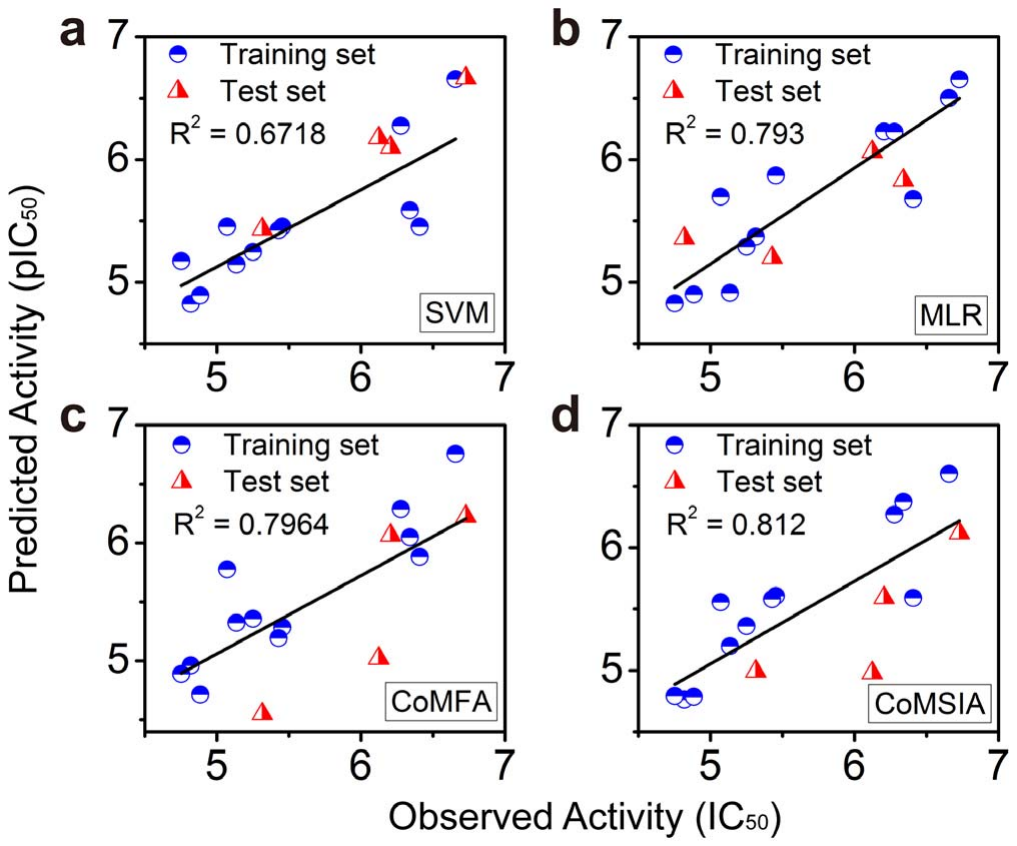
Ligand-Based and QSAR model validation. Coefficient correlations (R2) were used to validate the predicted model. (a) The linear model shows R^2^ of 0.6718 and (b) the nonlinear model shows a promising R^2^ of 0.793. (c–d) High coefficient correlation (In biomedical criteria, R^2^ > 0.5 represents a predictable model) of QSAR model suggests QSAR is more suitable in predicting UT-B inhibitor bioactivity.

The aforementioned descriptors were then used in the generation of SVM and MLR models. The R^2^ of the SVM and MLR models were 0.6718 and 0.793 (Figure 6a, b), which suggests that the model of SVM is acceptable (>0.5). The model of MLR is suitable for prediction (~0.8). The non-QSAR predicted pIC_50_ of the UT-B inhibitors are listed in Table 5. The residuals of pIC_50_ of PU_21_, PU_168_, PU_468_ and PU_474_ are: 1.442, 0.274, 0.726 and 0.869, respectively. Compared with the SVM model, MLR shows more promise in calculating the prediction set. Descriptors in GFA, such as number of HA, MS, MPSA, and MPSASA, have contributed greatly to the stabilization of protein-ligand interaction. Moreover, HA has contributed strongly in defining drug-like properties *in silico*. The number of hydrogen bond acceptors of PU_21_, PU_168_, PU_468_ and PU_474_ are: 4, 6, 3, and 6, respectively. However, the binding affinities of PU_168_ and PU_474_ are restrained by TRP286.

Quantitative structure activity relationship (QSAR) was constructed by CoMFA and CoMSIA. The PLS results are listed in Table 6. The CoMFA models, in which the steric file was used as a primary parameter, were constructed with an optimal number of components (ONC) of six, and with cross validation and non-cross validation correlation coefficients q^2^ and r^2^ of 0.682 and 0.796, and with an F-test value of 32.57. For the CoMSIA model, steric, hydrophobic, and h-bond acceptor properties were coupled with a model ONC of six, with the highest r^2^ and q^2^, of 0.812 and 0.676 respectively.

**Figure 7:**
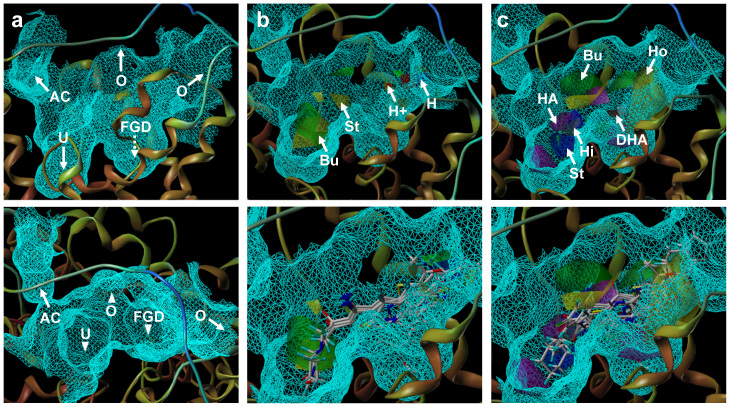
(a) Side view (top) and top view (bottom) of UT-BI binding site. AC represents allosteric channel, U represents urea binding site, FGD represents functional group docking (FGD) site, O represents the opening of binding site. (b) CoMFA contour maps, four contours represent steric favored contour (St, yellow), Bulk favored contour (Bu, green), electrostatics favored contour (H^+^, blue) and Electrostatics disfavored contour (H, red). (c) CoMSIA contour maps, six contours represent hydrogen bond acceptor favored contour (HA, magenta) and disfavored contour (DHA, red), hydrophobic favored contours (Ho, yellow) and hydrophobic disfavored contours (Hi, white), steric favored contour (St, blue) and bulk favored contour (Bu, green). The cartoon graph of CoMSIA illustrates the contour location inside the UT-BI binding site.

The chosen CoMFA and CoMSIA models were used to generate a QSAR contour map (Figure 7). The binding pocket of UT-B inhibitors can be primary divided into three parts: urea channel (U), functional group docking (FGD) site (containing TRP286 and ASN289) and allosteric channel (AC) (Figure 7a). The original hypothesis is that FGD provides a pocket for UT-B inhibitor binding. However, only PU_21_ can anchor to FGD. Compared to the contour fields, the steric favored contours (green) are anchored near the UT-B-binding site, whereas the bulk favored contours (green) are close to the UT-B inhibitor binding sites (Figure 7b). Electrostatics (H^+^) that favored contours (blue) are located near the opening of the binding site (O). No contour is located in AC and FGD. The CoMSIA contour map shows a hydrogen bond acceptor (HA) favored contour (magenta) is located near FGD, that matches the functional group properties of PU_21_ and allows PU_21_ to form hydrogen bonds to TRP286 and ASN289 (Figure 7c). Hydrophobic (Ho) favored contours (yellow) and hydrophobic disfavored contours (white) are located near the terminals of the inhibitor binding site. The correlation coefficients (R^2^) are 0.796 and 0.812 for CoMFA and CoMSIA models (Figure 6c, d), respectively, which indicates suitable bioactivity prediction. The range of residuals between observed pIC_50_ and predicted pIC_50_ is < ± 1.2.

### Molecular dynamic simulation

**Figure 8:**
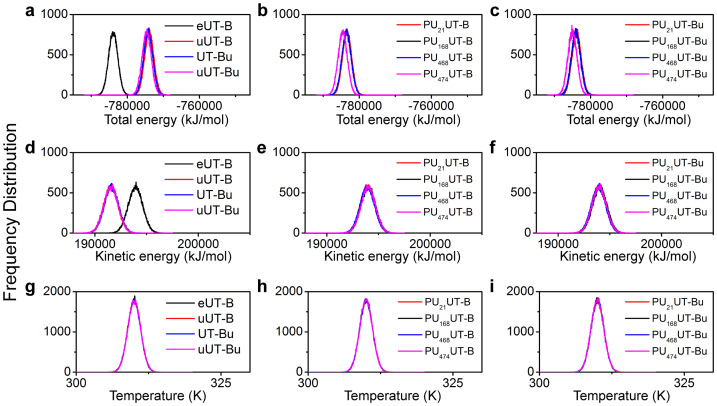
Physical properties of the molecular dynamics environment. (a–c) Total energy, (d–f) kinetic energy and (g–i) temperature were calculated. All complexes were performed at constant temperature. Total energy and kinetic energy shows the stability of the system. UT-B without ligand shows a relatively stable system compared to urea. Total energy decreased while the ligand was binding with UT-B, either in urea treated and untreated conditions.

**Figure 9:**
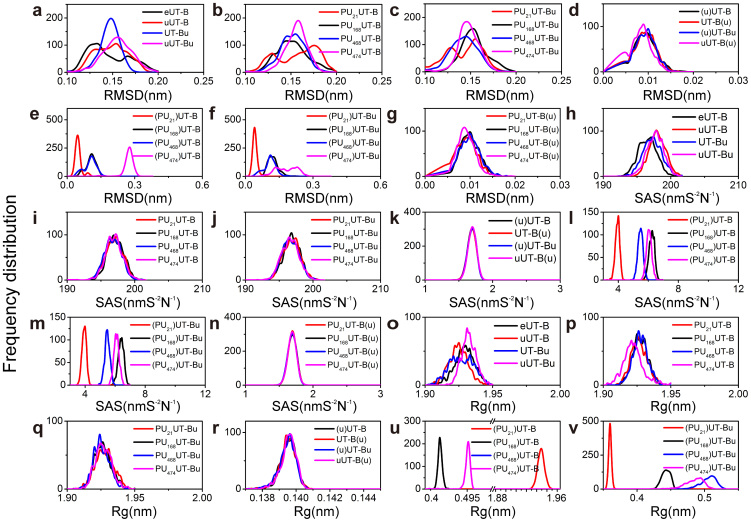
(a–g) Root-mean-square deviation (RMSD) analysis of UT-B complexes. (h–n) Solvent accessible surface area (SAS) analysis of UT-B complexes. (o–v) Radius of gyration (Rg) analysis of UT-B complexes. By calculating the distribution of RMSD, we can estimate the conformational changing frequency of UT-B compared with UT-B initial state. Calculating SAS provides interaction detail between solvent and molecule, which reflects the changes in the binding site surface area. The radius of gyration was often used to measure protein density.

The mean root-mean-square deviation (RMSD) of eUT-B (video 1) is 0.143 nm. The eUT-B shows the lowest total energy of mean −783,917 KJ/mol (Figure 8a) and the highest kinetic energy of 193,919 KJ/mol (Figure 8d). Compared to the 0.146 nm of uUT-B (video 2), 0.144 nm of UT-Bu (video 3) and 0.153 nm of uUT-Bu, although the extracellular urea leaves the urea binding site after 2 ns (video 4), eUT-B is relatively stable (Figure 9a). When two urea exist, the UT-B is even more unstable than a single UT-B (Figure 9d). There is no significant differences between the mean RMSD of the inhibitor bound and inhibitor unbound urea combined at the intracellular binding site (Figure 9b, c). Therefore the function of urea at this binding site cannot be proved by RMSD. PU_21_ has a higher stability in PU_21_UT-B (video 5) and PU_21_UT-Bu (video 6), but the UT-B of PU_21_ in unbound urea has a higher radius of gyration (mean Rg > 1.9 nm) (Figure 9p). In PU_21_ UT-Bu, Rg of PU_21_ reduces to below 0.2 nm (Figure 9q). Therefore, when urea is lacking, PU_21_ tends to self-unfold. Furthermore, the relation between PU_21_ and solvent with water molecules in bound and unbound UT-B are relatively low (Figure 9l, m) in which the solvent accessible surface area (SAS) is 3.920 and 3.912 nm S^−2^N^−1^, respectively. This proves that a binding site of PU_21_ can be surrounded by FGD and lower the reaction surface between PU_21_ and the water molecules.

**Figure 10:**
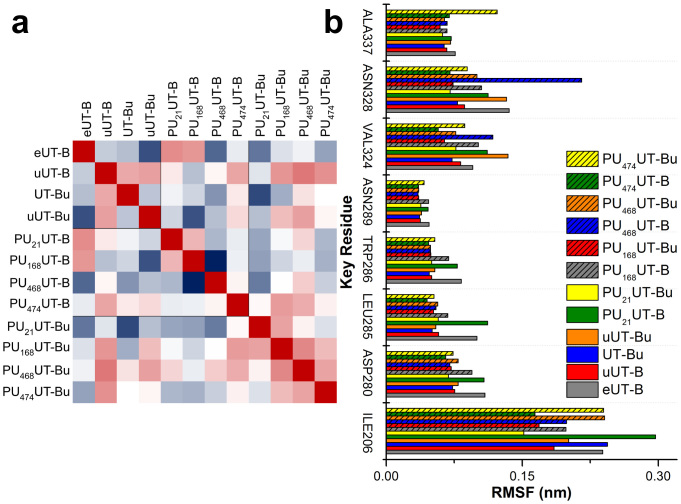
(a) Heat map of root mean square fluctuation (RMSF) of UT-B complexes. The correlation between UT-B complexes were calculated and ranked by coefficient correlation (R2), in which the highest R2 is 1 (red), medium is white and the lowest is presented in blue. (b) RMSF of UT-B key residues in each complex, ALA337, ASN328, VAL324, ASN289, TRP286, LEU285, ASP280 and ILE206 are shown. The individual values contained in a matrix were used to demonstrate how inhibitors influence UT-B and estimate the common mechanism.

Root mean square fluctuations (RMSF) were calculated to study the stability of individual residues in independent systems. The correlations between independent systems were then calculated to find functional changes caused by system differences (Figure 10). The urea in complex uUT-B has the functions of urea in UT-Bu and uUT-Bu (Figure 10a). However, the correlation coefficient (R^2^ = 0.6848) between UT-Bu and uUT-Bu is lower. The overall uUT-B mean RMSF is 0.0790 nm, which is lower than the 0.0811 nm of UT-Bu. This indicates that the urea located at the extracellular binding site is far more able to stabilize the UT-B structure than intracellular urea urea. A high RMSF correlation exists between eUT-B and PU_168_UT-B (video 7) as well as PU_21_UT-B, which are R^2^ = 0.8417 and R^2^ = 0.852, respectively. A high degree of correlation is produced between PU_168_UT-Bu (video 8) and uUT-B as well as uUT-Bu (R^2^ = 0.842, R^2^ = 0.801). PU_474_UT-B (video 9), on the other hand, has a high level of correlation between uUT-B and uUT-Bu; R^2^ = 0.826 and R^2^ = 0.770, respectively. This indicates that PU_474_UT-B has a similar effect to urea in uUT-B and produces a similar induced-fit mechanism. PU_168_ being at the intracellular urea bounded UT-B, causes PU_168_ to produce the same functions as the urea of uUT-B (R^2^ = 0.842) in the PU_168_UT-Bu. There is a very high mutual correlation between PU_168_UT-Bu, PU_468_UT-Bu (video 10), and PU_474_UT-Bu, whereas PU_21_UT-Bu only produces a high correlation with PU_168_UT-Bu (R^2^ = 0.829), with molecular mechanisms similar to, but not the same as, those of PU_168_. On the contrary, PU_21_UT-Bu is different from all urea bound UT-B and inhibitor bound UT-B (RMSF = 0.0835 nm). Therefore, it can be inferred that the inhibitor produces different mechanisms in PU_21_UT-Bu, and the urea in PU_21_UT-Bu may induce this change in mechanism to occur, making conformation of PU_21_UT-B transform.

**Figure 11:**
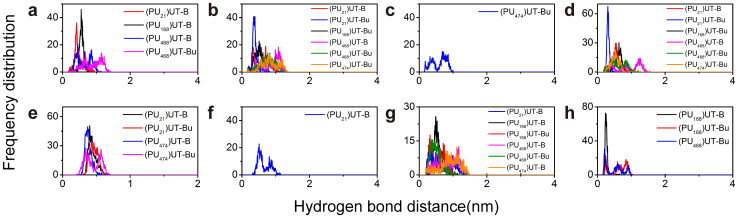
Hydrogen bond distance measurement between UT-B-inhibitors and UT-B, residues include (a) ILE206, (b) ASP280, (c) LEU285, (d) TRP286, (e) ASN 289, (f) VAL324, (g) ASN328 and (h) ALA337. The cutoff distance of hydrogen bond generation was set to 0.35 nm.

ILE206 reacts with PU_168_UT-B, PU_21_UT-B and PU_468_UT-B (video 11) and produces hydrogen bonds (Figure 11a). The hydrogen bonds produced by PU_21_ have the highest frequency (9%) (Table 7). The RMSF of ILE206 in PU_21_UT-B is 0.297 nm, which is the highest in all complexes (Figure 10b). ASP280 participates in inter-reactions with complex UT-B inhibitor other than PU_168_UT-B and PU_474_UT-B. The PU_21_UT-Bu hydrogen bond frequency is the highest (33.9%) and the occurrence frequency of 21.5% of PU_21_UT-B is the second highest (Figure 11b). ASP280 RMSF is highest with eUT-B and PU_21_UT-B, being 0.109 nm and 0.108 nm, respectively (Figure 10b). TRP286 participates in hydrogen bond formation between PU_21_UT-Bu and PU_21_UT-B with 83.9% and 3.1% occurrence rates, and also participates in hydrogen bond formation between PU_474_UT-B and PU_468_UT-B (11% and 5.1%) (Figure 11d). The RMSF of TRP286 eUT-B and PU_21_UT-B are 0.083 nm and 0.078 nm. The RMSF of ASN328 in PU_468_UT-B is 0.216 nm, as well as 0.1 nm and 0.136 nm in PU_468_UT-Bu and eUT-B (Figure 10b). ALA337 forms hydrogen bonds with PU_168_UT-B and PU_168_UT-Bu, which are 56.5% and 15%, respectively (Figure 10h). In addition, ALA337 also forms hydrogen bonds with PU_474_UT-Bu (9%), the RMSF in PU_478_UT-Bu (video 12) also being the highest (0.1224 nm).

## Discussion

Comparing RMSF with the UT-B complex key residue using eUT-B as the control group shows that besides PU_21_UT-B, all the others have a trend of reduction (Figure 10b). Comparing the correlation between PU_21_UT-B and PU_21_UT-Bu using the above method, it was found that PU_21_ was not able to bind the extracellular urea binding site (correlation of eUT-B, R^2^ = 0.852) like the other three UT-B inhibitors, and that the other UT-B inhibitors can simulate urea (Figure 10a). The PU_21_ radius of gyration is larger than 1.9 nm, whereas the solvent accessible surface area is lower than 4.0 nmS^−2^N^−1^. Therefore, the PU_21_ function group can bind to FGD, which provides protection as well as allowing the function group to produce more bonds using the advantage of the radius of gyration. On the other hand, results suggest that PU_168_, PU_468_ and PU_474_ can perform a similar role as extracellular urea. Although in the W286G mutation model, the binding affinity of both PU_168_ and PU_474_ increases, they participate in the formation of hydrogen bonds in a stable manner in molecular dynamics simulation. Residues that participate in the formation of PU_21_ hydrogen bonds include ASP280, ASN289, LEU285, and VAL324, none of them can reduce inhibitor binding stability. In W286A mutation tests, the bonding strength between PU_21_ and UT-B drops from 46.437 to 43.198. In W286G, the bonding strength of PU_21_ drops to 42.752. In the D280A test, the PU_21_ bonding strength drops to 43.549. Therefore, it can be inferred that ASP280 and TRP286 can both increase and decrease inhibitor binding stability.

**Figure 12:**
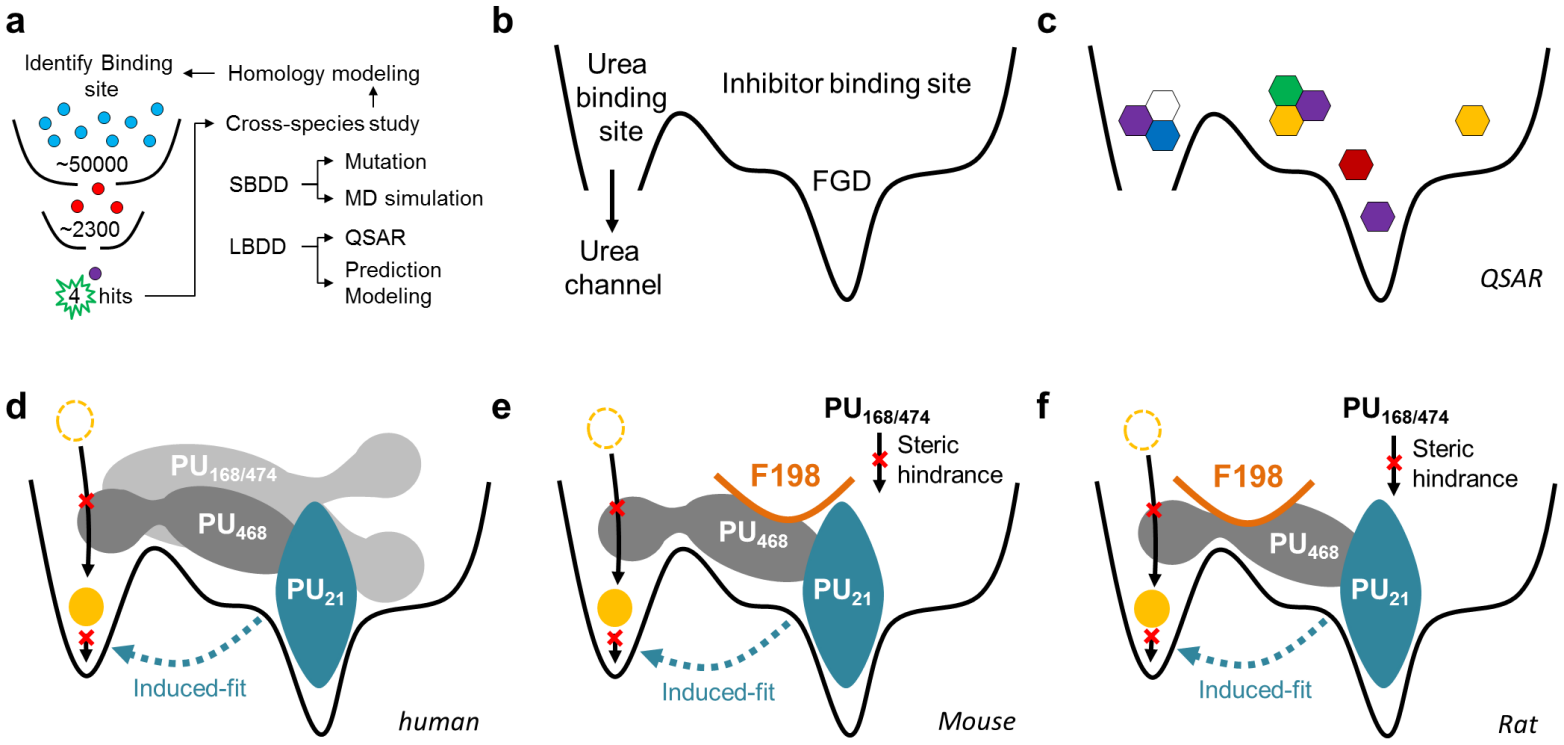
(a) Flow chart of this investigation. A small molecule database is employed to identify UT-B inhibitors via HTVS and high-throughput screening. (b) Inhibitors were used to map novel inhibitor binding sites by *in silico* methods. Inhibitor binding site was found to overlap a part of the urea binding site. A cryptic binding pocket, such as FGD, was discovered to be important in anchoring an inhibitor that contains residues TRP286 and ASN289. (c) Quantitative structure activity relationship suggested a hydrogen bond acceptor favored property is located near FGD which provides proper interaction pocket to PU_21_. The inhibition mechanism hypothesis was supported by fine-grained molecular dynamics. (d) All atom simulations suggest small inhibitors, such as PU_21_, might generate induced-fit mechanisms in urea transportation blocking. By generating steric hindrance directly towards urea binding site, other larger inhibitors are able to generate inhibition activity similar to PU_21_. This cross-species study discovered that the migration pathway of PU_168_ and PU_474_ might be interrupted by residue PHE198 in either (e) mouse or (f) rat model.

In conclusion, computational modeling was primarily used to predict inhibitor binding sites. Four UT-B inhibitors; PU_21_, PU_168_, PU_468_ and PU_474_ were identified via HTVS and high throughput screening by using an erythrocyte osmotic lysis assay and *in silico* methods (Figure 12a). By integrating structure-based and QSAR, potential cryptic binding pockets, such as FGD, were discovered that could be important in anchoring an inhibitor (Figure 12b–c). The species comparison study discovered inhibitory activity differences between human, rat and mouse UT-B (Figure 12d–f). Binding affinity calculation suggests that PHE198 might block the inhibitor migration pathway, leading to a decrease in inhibitory activity. Molecular dynamics simulation provided evidence of an inhibitor binding mechanism. Predominately, PU_168_, PU_468_ and PU_474_ were predicted to exhibit a similar induced-fit mechanism of urea in the urea binding site. PU_21_ likely produced a remarkable anchoring function in the UT-B FGD domain, in both the PU_21_UT-B and PU_21_UT-Bu complex system. Moreover, key residues including ASP280, TRP286 and ASN289 were identified by a structure-based study, and were double validated by simulation and *in silico* mutation studies. This pioneer study provides a structural basis for future lead identification and optimization.

## Methods

### High-Throughput Virtual Screening

UT-B protein structure used in docking was downloaded from Protein Data Bank (PDB: 4EZD)^19^. Human UT-B was then input to generate a homology model^22^ by using *Bos taurus* (Bovine) UT-B sequence as a template (Swissprot entry: Q13336. Species: human^23^). BLOSUM^24^ was chosen to be used as a multiple alignment scoring matrix with a 10 gap open penalty. Validation server RAMPAGE was used to verify validity of the predicted model^25^,^26^. The urea binding sites in human UT-B were then defined and side chains were also optimized. Three hypothetical sites were predicted to filter the 50,000 small-molecular drug-like compounds from the Asinex database, one of these hypothetical sites was later evaluated by UT-B inhibitors.

### Compounds for screening

50,000 small-molecular drug-like compounds were screened from a database and 2,319 compounds were identified and purchased from a commercial chemical company (Asinex, Russia). These 2319 compounds were resolved and stored in 10 mM in dimethyl sulfoxide (DMSO).

### Collection of human, rat, and mouse blood

Vein blood was collected from humans, SD rats, wild-type mice or UT-B-null mice as described previously [21].

### UT-B Inhibitor Identification by High-Throughput Screening

High-throughput screening assay was performed for identification of UT-B inhibitors using erythrocytes, which originally express UT-B as described previously^27^. Erythrocytes were diluted to a hematocrit value of 2% in hyperosmolar PBS containing 1.25 M acetamide and 5 mM glucose. Erythrocyte suspensions were preserved at room temperature for 2 h by periodic pipette mixture. Then, 99 μl erythrocyte suspension from a reservoir was added to each well of a 96-well round-bottom microplate, to which test compounds were added (1 μl, 10 μM final compound concentration, 1% final DMSO concentration). After 6 min of incubation, 20 μl of the erythrocyte suspension was added rapidly to each well of a 96-well black-walled plate containing 180 μl isomolar buffer (PBS containing 1% DMSO) in each well. Erythrocyte lysis was quantified from a single time point measure of absorbance at 710 nm wavelength with a plate reader (BioTek)^27^.

The percentage of erythrocyte lysis in each test well was calculated using control values from the same plate as: % lysis = 100%. (A_neg_-A_test_)/(A_neg_-A_pos_), where A_test_ is the absorbance value from a test well. Nonspecific UT-B inhibitor phloretin (Sigma-Aldrich, 700 μM final concentration) was added as an additional positive control.

### Structure-based drug design

Twenty compounds were prepared for a Monte Carlo docking simulation^28^. Force field CHARMm^29^,^30^ was employed to start minimization. A receptor-rigid docking algorithm (LigandFit) was employed to calculate ligand binding affinity, in which minimized docking poses were then clustered with 1.5 RMS Threshold for Diversity^31^. Scoring functions such as the potential of mean force (PMF)^32^, Jain and Piecewise Linear Potential 1/2 (PLP1/2)^33^ were used to validate the major determinate-Dock Score (Dock Score = - ligand/receptor interaction energy + ligand internal energy). A mutation study was employed to evaluate the role of key residues by generating seven mutation models that were used for further re-docking^34^. Results were further used to produce a scaffold for molecular dynamics simulation.

### Ligand-based drug design

Activity was predicted using QSAR models^35^. This study establishes activity prediction models for Ligand prediction using compounds with UT-B inhibitory activity screened by the erythrocyte osmotic lysis assay. The chemical properties for the compounds were calculated through DS 2.5, and more than 200 descriptors were produced. Then, genetic function approximation (GFA) was used to filter out and select descriptors with higher relevance, and the square correlation coefficient (r2) was used for ordering and selection^36^. Training sets and test sets were obtained by random allocation of the descriptors selected through GFA (Table S1) along with the IC_50_s of the sixteen analogues of PU_21 _[Ren et al., under review]. Support Vector Machine (SVM) and Multiple Linear Regression (MLR)^37^, respectively, used LibSVM^38^ and MATLAB (MATrix LABoratory, Natick, MA, US: The MathWorks Inc.) to establish linear and non-linear models. 3D-QSAR models were established to understand the structural characteristics of the compounds. Comparative force field analysis (CoMFA) was used to study steric and electrostatic properties. Comparative similarity indices analysis (CoMSIA) was used to study steric, electrostatic, hydrophobic, hydrogen donor and acceptor properties. The training set and test set used the atom-fit module of SYBYL-X 1.1 (St Louis, M. USA: Tripos.) to conduct scaffold alignment. Then, CoMFA and CoMSIA models were established. Coulombic potential and Lennard-Jones potential (LJP) were used to calculate electrostatic fields and steric fields, respectively. Gaussian functions were used to calculate steric, hydrogen bond acceptor and donor, hydrophobic and electrostatic fields in the CoMSIA model. From the results of the partial least squares (PLS) analysis, the conventional correlation coefficient (r2) and cross-validated coefficient (q2) were produced, which were used to evaluate the accuracy of non-cross validation and cross validation models, respectively^39^. All tested SVM, MLR, and 3D-QSAR models were used to predict the activity of the predicted sets.

### Molecular Dynamics

**Figure 13:**
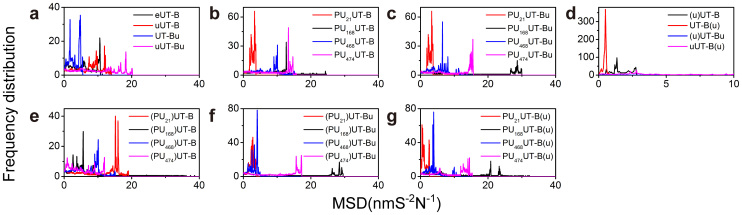
Mean square displacement (MSD) of UT-B complexes and ligands that measure the random motion of urea, ligand and UT-B. The MSD determines how the UT-B complex diffuses in the solvent system.

The experiment was divided into three portions that simulated the structural and dynamic differences of UT-B under normal conditions when inhibitors exist. Unbound UT-B (eUT-B), UT-B bound with single urea (uUT-B and UT-Bu, in which uUT-B represents urea binding in the extracellular binding site close to the inhibitor binding site), and UT-B bound with double urea (uUT-Bu) were used to simulate UT-B states under normal functionality. Four systems were used to simulate the effects of inhibitors in UT-B (PU_21_, PU_168_, PU_468_ and PU_474_). In addition, four systems were used to simulate the situation in which urea and inhibitors both exist in UT-B (PU_21_UT-Bu, PU_168_UT-Bu, PU_468_UT-Bu, PU_474_UT-Bu). Force field CHARMm27^40^ and parameters were added to each ligand by using SwissParam^41^ and the pdb2gmx protocol of Gromacs version 4.5^42^. Coupled ligand-UT-B complex and uUT-Bu complex, ligand-UT-B-urea complex were used to generate a cubic box and to immerse into a buffer solution (solvated with TIP3P water model)^43^,^44^. Mean square of displacement (MSD) was used to demonstrate the diffusion of molecules in the system (Figure 13). The distance between the edge of the cubic box and complex was set to 1.2 nm. 0.145 M NaCl ions were added to neutralize the system. A steepest descent algorithm was calculated for minimization, and minimization would stop when *max(|Fn|)* < *ε* or defined minimization steps had been approached. Maximum steepest descents minimization was set to 5,000 time steps. For the equilibration, the last configuration of energy-minimization was used to generate restrained dynamics production. NVT equilibration, Particle-Mesh Ewald (PME) and Berendsen weak thermal coupling methods were used in dynamics production, whereas PME was also used in the calculation of electrostatic interactions. The time step was set at 2 fs under the PME option, where the cut-off for PME was 1.0 nm. Simulation trajectories analysis was conducted by plug-in open source methods.

### Ethics Statement

All experiments were carried out in accordance with the Regulations for the Administration of Affairs Concerning Experimental Animals of Peking University. The experimental protocol was approved by ethics committee of Peking University. For experiments with human erythrocytes, the informed consent was obtained from all subjects.

## Supplementary Material

Supplementary Information video 1

Supplementary Information video 2

Supplementary Information video 3

Supplementary Information video 4

Supplementary Information video 5

Supplementary Information video 6

Supplementary Information video 7

Supplementary Information video 8

Supplementary Information video 9

Supplementary Information video 10

Supplementary Information video 11

Supplementary Information video 12

Supplementary Information Supplementary file

## Figures and Tables

**Table 1:** UT-B inhibition potency of four compounds in three species

	Structure	IC_50_ (μM)		
Human	Rat	Mouse		
**PU_21_**		3.78 ± 0.48	2.62 ± 0.12	4.81 ± 0.40
**PU_168_**		0.20 ± 0.03	>	>
**PU_468_**		5.63 ± 0.82	3.18 ± 0.24	4.13 ± 0.33
**PU_474_**		0.28 ± 0.01	>	>

“>” indicates the IC_50_ of analogs was greater than 10 μM.

**Table 2:** The docking results of UT-B inhibitors

Name	pIC_50_	Dock Score	-PLP1	-PLP2	Jain	-PMF	Rotate bond	MW	Internal energy
PU-02	5.071	41.432	57.71	51.95	0.39	90.13	1	256.32	−2.507
PU-14	5.454	42.849	56.93	54.92	0.86	93.68	1	256.32	−3.163
PU-23	5.429	48.055	58.07	54.7	0.6	90.91	3	286.35	−3.933
PU-26	6.206	64.201	78.7	75.14	2.84	128.09	4	452.55	−5.608
PU-31	6.408	41.137	59.24	52.45	0.7	87.59	1	256.32	−2.371
PU-33	5.250	62.756	79.33	70.25	1.32	109.36	4	368.47	−4.888
PU-34	5.136	41.824	56.64	54.41	1.46	91.23	1	257.31	−2.631
PU-35	6.339	58.734	78.47	72.11	2.27	114.11	3	341.43	−4.895
PU-47	4.819	58.649	81.74	72.78	1.64	121.64	4	357.43	−3.651
PU-48	6.728	47.185	60.03	57.82	1.02	90.92	3	288.32	−3.872
PU-49	4.885	61.726	74.8	72.58	1.63	115.3	5	381.42	−6.483
PU-55	6.657	49.098	63.3	56.8	0.11	98.52	4	302.35	−4.635
PU-57	4.753	55.203	73.65	67.08	1.34	118.49	3	369.48	−3.693
PU-58	6.123	60.2	71.81	65.8	1.5	123.37	5	370.47	−5.177
PU-59	6.276	27.012	50.23	48.35	−0.35	104.38	6	532.58	13.1
PU_21_	6.770	46.437	71.67	64.9	2.55	86.75	2	272.32	−2.516
PU_168_	6.553	19.037	81.77	72.01	4.25	117.94	8	472.59	8.399
PU_468_	6.167	50.314	60.47	49.37	1.63	116.07	4	393.83	−1.896
PU_474_	6.495	58.53	86.32	79.38	2.9	124.9	7	451.5	0.273

pIC_50_ = -log(IC_50_) + 6.

**Table 3:** Re-docking result of four compounds in three species

	Rat		Mouse	
Name	Dock Score	Residual	Dock Score	Residual
PU_21_	62.118	15.681	62.308	15.871
PU_168_	43.655	24.618	53.446	34.409
PU_468_	64.505	14.191	72.361	22.047
PU_474_	59.897	1.367	61.178	2.648

Residual = Score(Rat or Mouse) – Score (human).

**Table 4:** Superimposed and root mean square deviation (Å) evaluation of UT-B in four species

Species	Bovine	Mouse	Rat	Human
**Bovine**	0	0.1429	0.1227	0.1352
**Mouse**	0.1429	0	0.1176	0.1411
**Rat**	0.1227	0.1176	0	0.1114
**Human**	0.1352	0.1411	0.1114	0

**Table 5:** SVM, MLR and QSAR predicted model

			SVM		MLR		CoMFA		CoMSIA	
Index	Name	pIC_50_	Predict	Residual	Predict	Residual	Predict	Residual	Predict	Residual
1	PU-02	5.071	5.454	−0.383	5.697	−0.626	5.777	−0.706	5.556	−0.485
2	PU-14	5.454	5.454	0	5.870	−0.416	5.286	0.168	5.606	−0.152
3	PU-23	5.429	5.425	0.004	*5.201	0.228	5.193	0.236	5.581	−0.152
4	PU-26	6.206	*6.100	0.106	6.228	−0.022	*6.025	0.181	*5.592	0.614
5	PU-31	6.408	5.454	0.954	5.679	0.729	5.883	0.525	5.591	0.817
6	PU-33	5.250	5.246	0.004	5.289	−0.039	5.357	−0.107	5.362	−0.112
7	PU-34	5.136	5.144	−0.008	4.913	0.223	5.323	−0.187	5.198	−0.062
8	PU-35	6.339	5.587	0.752	*5.831	0.508	6.049	0.29	6.374	−0.035
9	PU-47	4.819	4.824	−0.005	*5.359	−0.54	4.961	−0.142	4.762	0.057
10	PU-48	6.728	*6.664	0.064	6.654	0.074	*6.226	0.502	*6.12	0.608
11	PU-49	4.885	4.894	−0.009	4.902	−0.017	4.715	0.17	4.784	0.101
12	PU-55	6.657	6.656	0.001	6.501	0.156	6.755	−0.098	6.602	0.055
13	PU-57	4.753	5.174	−0.421	4.828	−0.075	4.89	−0.137	4.79	−0.037
14	PU-58	6.123	*6.177	−0.054	*6.063	0.06	*5.022	1.101	*4.98	1.143
15	PU-59	6.276	6.276	0	6.227	0.049	6.287	−0.011	6.27	0.006
**16	PU_21_	6.770	5.328	1.442	6.385	0.385	6.161	0.609	7.027	−0.257
**17	PU_168_	6.553	6.279	0.274	6.515	0.038	6.186	0.367	6.667	−0.114
**18	PU_468_	6.167	5.441	0.726	6.042	0.125	4.974	1.193	5.027	1.14
**19	PU_474_	6.495	5.626	0.869	6.639	−0.144	6.238	0.257	6.759	−0.264

*:Test set.

**:Prediction set.

**Table 6:** The evaluation of QSAR models which were constructed by a partial least squares (PLS) algorithm

			Cross validation		Non-cross validation			Fraction				
	CoMFA*	CoMSIA	ONC	q^2^	r^2^	SEE	F	S	E	H	D	A
**ONC**	6	S	6	0.596	0.702	0.559	19.63	1.000		-	-	-
**q^2^cv**	0.682	H	6	0.544	0.784	0.475	30.31	-	-	1.000		-
**r^2^**	0.796	D	6	0.454	0.523	0.707	9.13	-	-	-	1.000	
**SEE**	0.462	A	6	0.531	0.756	0.506	25.78	-	-	-	-	1.000
**F**	32.57	SH	6	0.525	0.807	0.450	34.85	0.376	-	0.624	-	-
		SD	6	0.503	0.701	0.560	19.50	0.627	-	-	0.373	-
		SA	6	0.578	0.785	0.475	30.41	0.436	-	-	-	0.564
		HD	6	0.512	0.769	0.492	27.70	-	-	0.666	0.334	-
		HA	6	0.562	0.802	0.455	33.78	-	-	0.547	-	0.453
		DA	6	0.499	0.808	0.449	35.00	-	-	-	0.312	0.688
		SHD	6	0.638	0.776	0.484	28.90	0.209	-	0.514	0.276	-
		SHA*	6	0.676	0.812	0.444	35.99	0.235	-	0.434	-	0.331
		SDA	6	0.623	0.765	0.496	27.15	0.324	-	-	0.266	0.410
		HDA	6	0.624	0.773	0.487	28.43	-	-	0.445	0.252	0.302
		SEHD	6	0.652	0.776	0.484	28.90	0.209	0.000	0.514	0.276	-
		SEHA	6	0.673	0.812	0.444	35.99	0.235	0.000	0.434	-	0.331
		SEDA	6	0.631	0.765	0.496	27.15	0.324	0.000	-	0.266	0.410
		SHDA	6	0.625	0.777	0.483	29.05	0.163	-	0.328	0.231	0.279
		EHDA	6	0.627	0.773	0.773	28.43	-	0.000	0.445	0.252	0.302
		SEHDA	6	0.634	0.777	0.483	29.05	0.163	0.000	0.328	0.231	0.279

The above abbreviations represent:

A: Acceptor. D: Donor. E: Electrostatic. F: F-test value. H: Hydrophobic. ONC: Optimal number of components. PLS: partial least squares. S: Steric. SEE: Standard error of estimate. *: Optimum prediction model.

**Table 7:** Hydrogen bond percentage calculated between UT-B inhibitor and UT-B

	Key Residue							
	ILE206	ASP280	LEU285	TRP286	ASN289	VAL324	ASN328	ALA337
**(PU_21_)UT-B**	9	21.5	0	3.1	2.9	0.5	17.3	0
**(PU_168_)UT-B**	0.8	0	0	0	0	0	2	56.5
**(PU_468_)UT-B**	5.1	1.9	0	5.1	0	0	3.7	0
**(PU_474_)UT-B**	0	0	12.5	11	30.3	0	5	0
**(PU_21_)UT-Bu**	0	33.9	0	83.9	1.3	0	0	0
**(PU_168_)UT-Bu**	0	10.9	0	6.5	0	0	22.7	15
**(PU_468_)UT-Bu**	0.6	1.3	0	3.9	0	0	17.1	0
**(PU_474_)UT-Bu**	0	0.2	0	0	27.7	0	1.4	9
