# Supplementary material for: Developing Hypothetical Inhibition Mechanism of Novel Urea Transporter B Inhibitor
Source: Sci Rep. 2014 Jul 22;4:5775. doi: 10.1038/srep05775 (PMC5376056; doi:10.1038/srep05775)
Supplement: Supplementary Information — Supplementary file [file srep05775-s13.pdf]

Developing Hypothetical Inhibition Mechanisms of Novel Urea Transporter B Inhibitor

Min Li [a, #], Weng Ieong Tou [b, #], Hong Zhou [a], Fei Li [c], Huiwen Ren [a], Calvin Yu-Chian Chen [b, d, e, f, g, *], Baoxue Yang [a,*]

[a] *The State Key Laboratory of Natural and Biomimetic Drugs, Department of Pharmacology, School of Basic Medical Sciences, Peking University, Beijing, 100191, China*

[b] *School of Medicine, College of Medicine, China Medical University, Taichung, 40402, Taiwan*

[c] *School of Pharmaceutical Sciences, Hubei University of Medicine, Shiyan, 442000, China*

[d] *Human Genetic Center, Department of Medical Research, China Medical University Hospital, Taichung, Taiwan*

[e] *Department of Biomedical Informatics, Asia University, Taichung, 41354, Taiwan*

[f] *Research Center for Chinese Medicine & Acupuncture, China Medical University, Taichung 40402, Taiwan*

[g] *Computational and Systems Biology, Massachusetts Institute of Technology, Cambridge, MA 02139, USA*

[#]*These authors contributed equally to this work*

* Corresponding Authors

Supplementary Table

Descriptors	Properties
ES_Sum_ssCH2	Sum descriptor for carbon with two single bonds
ES_Sum_aaCH	Sum descriptor for carbon with two aromatic bonds
ES_Sum_dssC	Sum descriptor for carbon with two single bonds and one double bond
ES_Sum_aasC	Sum descriptor for carbon with two aromatic bonds and one single bond
ES_Count_dssC	Sum descriptor for carbon with two single bonds and one double bond
Molecular_Solubility	logS, where S is the solubility in mol/L.
Num_H_Acceptors	Number of hydrogen bond acceptor
Molecular_PolarSurfaceArea	Calculates the polar surface area for each molecule using a 2D approximation.

Molecular_PolarSASA	Calculates the polar solvent accessible surface area for each molecule using a 2D approximation. The polar solvent accessible surface area is defined as the sum of the solvent accessible surface area of all the selected polar elements, which can include N, O, P, and S.
Energy	Gives the energy of the molecule's current 3D conformation evaluated using a variant of the published clean forcefield. It requires the molecules have 3D coordinates.

Supplementary Table 1. The definition of evaluated descriptors.
